# Acoustic, genetic and morphological variations within the katydid *Gampsocleis sedakovii* (Orthoptera, Tettigonioidea)

**DOI:** 10.3897/zookeys.529.6043

**Published:** 2015-10-26

**Authors:** Xue Zhang, Ming Wen, Junjian Li, Hui Zhu, Yinliang Wang, Bingzhong Ren

**Affiliations:** 1 Jilin Key Laboratory of Animal Resource Conservation and Utilization, School of Life Sciences, Northeast Normal University, Renmin St. 5268, Changchun 130024, P.R. China

**Keywords:** Acoustics, gene, morphology, subspecies, interim morphs

## Abstract

In an attempt to explain the variation within this species and clarify the subspecies classification, an analysis of the genetic, calling songs, and morphological variations within the species *Gampsocleis sedakovii* is presented from Inner Mongolia, China. Recordings were compared of the male calling songs and analysis performed of selected acoustic variables. This analysis is combined with sequencing of mtDNA - COI and examination of morphological traits to perform cluster analyses. The trees constructed from different datasets were structurally similar, bisecting the six geographical populations studied. Based on two large branches in the analysis, the species *Gampsocleis sedakovii* was partitioned into two subspecies, *Gampsocleis sedakovii sedakovii* (Fischer von Waldheim, 1846) and *Gampsocleis sedakovii obscura* (Walker, 1869). Comparing all the traits, the individual of Elunchun (ELC) was the intermediate type in this species according to the acoustic, genetic, and morphological characteristics. This study provides evidence for insect acoustic signal divergence and the process of subspeciation.

## Introduction

Acoustic signals are important in several social behaviors of insects, such as sexual selection (Derlink et al. 2014, Hirtenlehner and Römer 2014), predator defense (Kowalski et al. 2014), and species recognition (Marshall et al. 2008, Wikins et al. 2013). Most insects can make sounds using a variety of methods (Uvarov 1966). Members of the order Orthoptera, including katydids and crickets, utilize acoustic signals to communicate (Gray et al. 2014, Sarria et al. 2014). These signals, produced by the rubbing of a toothed vein on one wing against a plectrum on the other, results in songs by stridulation (Montealegre 2012, Robillard and Desutter-Grandcolas 2011).

*Gampsocleis* is a genus within Tettigoniidae, which includes sixteen species, eleven of which are found in China. *Gampsocleis sedakovii* (Fischer von Waldheim, 1846), a medium to large-sized, xerophilic, and slightly thermophilic katydid, is the most common and ubiquitous species distributed in northeast China. Individuals of *Gampsocleis sedakovii* are generally classified into two subspecies, *Gampsocleis sedakovii sedakovii* (Fischer von Waldheim, 1846) and *Gampsocleis sedakovii obscura* (Walker, 1869), differing morphologically in body size and the proportions of forewings and the pronotum (Zhou et al 2011).

The individuals of both subspecies (*Gampsocleis sedakovii sedakovii* and *Gampsocleis sedakovii obscura*) are excellent singers, and males sing at any time throughout the day. The calling song of *Gampsocleis sedakovii sedakovii* was already reported in a previous study (Wu and Shi 2009), but the acoustic signals of *Gampsocleis sedakovii obscura* have not been documented in the literature. Similarly, no comparative analysis of the songs from the two groups has been attained, which encouraged the development of this work.

The ratio between forewing and pronotum of *Gampsocleis sedakovii sedakovii* is much higher than that of *Gampsocleis sedakovii obscura*, while the *Gampsocleis sedakovii obscura* looks stronger than *Gampsocleis sedakovii sedakovii*. An “interim form” was found, consisting of individuals which had an intermediate ratio of forewing and pronotum between the averages for *Gampsocleis sedakovii obscura* and *Gampsocleis sedakovii sedakovii*, raising the possibility that the division of the subspecies within *Gampsocleis sedakovii* should be reconsidered (see also Rentz and Miller 1971).

Different insect species have different acoustic signals and these signals have been used as an invariable trait for the recognition of conspecifics and the discrimination of heterospecifics (Foster and Endler 1999). The interspecific specificity and intraspecific stability of insect songs are used as a significant index of classification (Montealegre-Z and Morris 2004, Hemp and Kehl 2010), although it remains difficult to distinguish cryptic species and subspecies. Sometimes the classification criteria for closely related species is unclear. Despite some molecular studies on *Gampsocleis sedakovii*, the relationship between these two subspecies and a clear basis of classification has remained controversial. Therefore, new methods to clarify these two subspecies and classify the interim morphs are required.

Wing polymorphism is common in insects, such as katydids (Wang 2011), grasshoppers (Steenman et al. 2015), rice planthoppers (Liang et al 2015), and so on. Three types of polymorphism are recognized: species with separate macropterous and brachypterous forms, continuous wing forms, and continuous wing form but with slightly reduced wing in the brachypterous form (Roháček 2012). The individuals of *Gampsocleis sedakovii* have continuous wings, and wing morph was often considered as a classification basis. Therefore, the wing types of *Gampsocleis sedakovii* were also examined to evaluate the differentiation of *Gampsocleis sedakovii* and its subspecies.

In this study the differentiation of the individuals collected from six locations of Inner Mongolia were analyzed and compared. Acoustic, morphological, and genetic differences were examined carefully. The analysis of the variation in the acoustic structure of *Gampsocleis sedakovii* from different geographical localities provided the basis for further explorations on the divergence on acoustic communication of this species and support the view that acoustic variation can promote the formation of subspecies.

## Methods

### Sound recording, tegmina measurement, and molecular sampling

**Figure 1.**
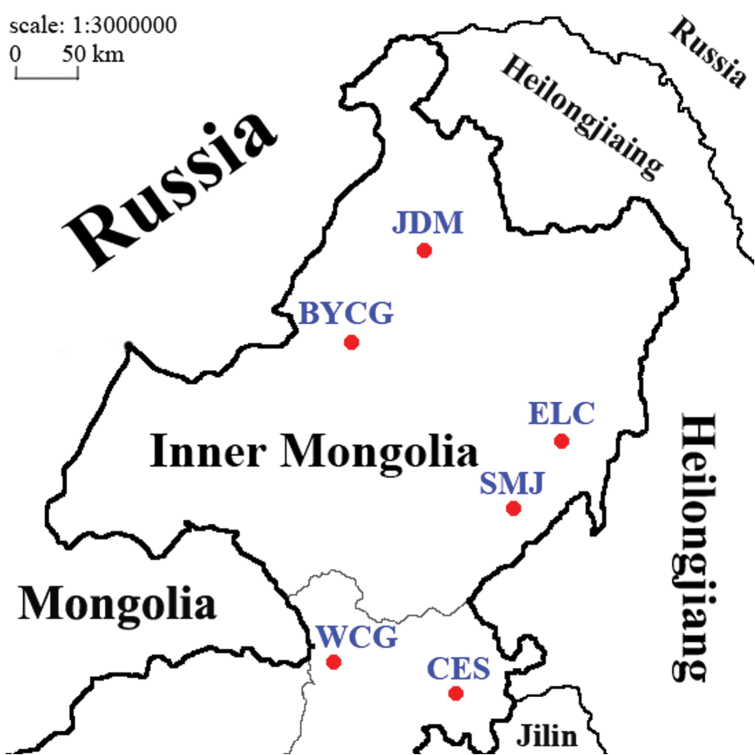
Locations of the six sampling sites in Inn Mongolia, China. Each point signifies a sampling site. Abbreviation: CES, Chaersen; BYCG, Bayancuogang; JDM, Jiaodaomu; WCG, Wuchagou; SMJ, Samajie; ELC, Elunchun.

In 2013, within 7 days, 40 adults were collected of *Gampsocleis sedakovii* from six localities in Inner Monglia, northeast China; individuals from CES (Chaersen), BYCG (Bayancuogang), JDM (Jiaodaomu), WCG (Wuchagou), SMJ (Shamajie), and ELC (Elunchun) were also used (Fig. 1). The number of calling individuals and the coordinates are shown in Table 1. Calling songs were recorded for each individual, with a digital voice recorder (PCM-D100 Digital Recorder, Sony Corporation, Tokyo, Japan) located at a distance of 20 cm from the singing insect (the distance was consistent). The sampling rate was 96 k-samples/s; S/N ratio was about 40 dB. It was previously reported that the acoustic behaviors and the traits of songs change with temperature (von Helversen 1972), so the environmental temperature for each sound file was recorded to ensure every record was collected within a certain range of ambient temperature.

**Table 1.** The number, geographic coordinates and total number of individuals sampled in acoustic analysis.

No.	Location	N	Longitude (E)	Latitude (N)
1	CES	8	121.9013°	46.4005°
2	BYCG	7	120.3006°	49.2014°
3	JDM	6	121.0001°	50.5005°
4	WCG	7	120.3021°	46.8003°
5	SMJ	6	122.1001°	47.6014°
6	ELC	6	122.4021°	48.2011°

Note: N means the number of samples. The abbreviations of the locations are shown in Figure 1 above.

Morphological structures (e.g., tegmina, pronotum, and body) were measured using 0.01 mm digital vernier calipers. The width of the stridulatory file teeth (WTSF) was measured under the scanning electron microscope (SEM) (JSM-6510LV, Hitachi Ltd, Tokyo, Japan), and the number of teeth in a stridulatory file (NTSF) were also counted under SEM. Forty individuals, whose songs had been recorded, were preserved in 70–95% ethanol solution for genetic analyses. Latitude, longitude, and sample number for each locality were also recorded (Table 1).

## Sound analysis

High quality sound samples were selected from all call sequences of each individual for acoustic parameters measurement using the software Cool Edit (Cool Edit pro V2.1, Adobe Systems). To remove the low frequency oscillations, high-pass filtering was performed before analysis. The cutoff frequency was 200 Hz. The song traits of these two subspecies were automatically analyzed using Matlab program (Matlab 7.0, Mathworks). The spectral analyses were also produced in Matlab using the toll Pwelch and the number of FFT points was 1024. The other parameters were set as default. The selected song traits were pulse duration (PD), pulse interval (PI), pulse repetition rate (PRR), dominant frequency (DF), highest frequency (HF), and lowest frequency (LF).

### Analysis of genetic differentiation

Cloning and sequencing of mitochondrial DNA control region within the genus *Gampsocleis* was previously conducted by Zhang, who found that *Gampsocleis sedakovii* haplotypes clustered into two distinct clades. Total genomic DNA was extracted from the hind femur muscles of 18 insects (selected from the samples obtained the acoustic data). DNA was extracted by a standard phenol-chloroform-isoamyl alcohol (PCI) extraction with slight modification (Sambrook et al. 1989). Amplification of the fragment was performed using the C1-J-1709 (AATTGGWGGWTTYGGAAAYTG) and C1-N-2353 (GCTCGTGTATCTACGTCTATWCC). Each PCR sample contained 5µl of 10 × PCR buffer at pH 8.3 (10 mmol/L of Tris-HCl at pH 8.3, 50 mmol/L KCL), 4 µl of 2.5 mmol/L MgCl_2_, 1.5 U of Taq DNA polymerase, 1 µl of 10 mmol/L of each deoxynucleotide triphosphate (dNTP) (C, G, A, T) all from Takara Biotech (Dalian, China), 2 µl of 10 µmol/L of each primer (Sangon Biotech, Shanghai, China), and 2 µl of DNA template and 33.7 µl ddH_2_O. The regions to be analyzed were amplified using standard PCR approaches with the following conditions: an initial denaturation at 94 °C for 3 min; 32 cycles at 94 °C for 30 sec, primer-specific annealing temperatures 55 °C for 30 sec, extension at 72 °C for 1 min; and final extension for 5 min at 72 °C. This resulted in the amplification of a fragment approximately 644 bp long. The amplicons were sequenced using a BigDye Terminator kit (Applied Biosystems) and an ABI 3730 automated sequencer (Applied Biosystems). Both sense and anti-sense strands were sequenced for all individuals.

### Cluster analysis

DNA sequences were aligned using the multiple-sequence program Clustal x 1.8 with parameters setting to default (Thompson et al. 1997). Phylogenetic analyses were performed by using MEGA version 6.0. Phylogenetic trees were reconstructed by neighbour-joinning (NJ).

Acoustic and stridulatory files characteristics of *Gampsocleis sedakovii*, obtained from specimens collected from different locations, were tested by cluster analysis using R Programming Language, respectively. Six traits were used in acoustic cluster analysis, including both aspects of time domain and frequency domain features: PD, PI, PRR, DF, HF, and LF. WL, NTSF, WTSF, LP, BL and WL/LP were contained in this analysis for morphological cluster.

## Results

### Calling Songs between individuals of different sampling sites

**Figure 2.**
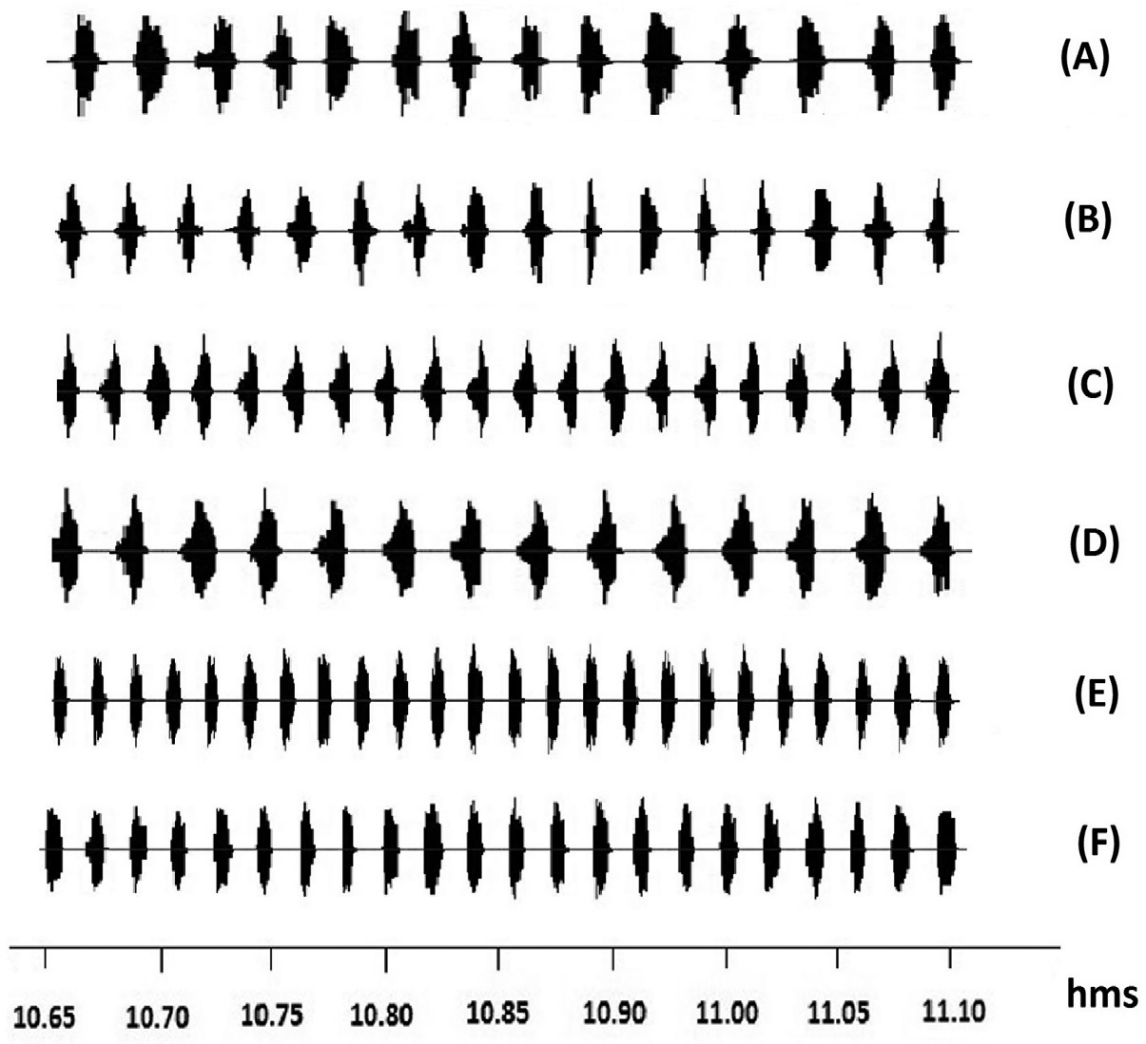
The oscillograms of calling songs of *Gampsocleis sedakovii* collected from six locations at different speeds (**A-F**: CES, BYCG, JDM, WCG, SMJ and ELC).

**Figure 3.**
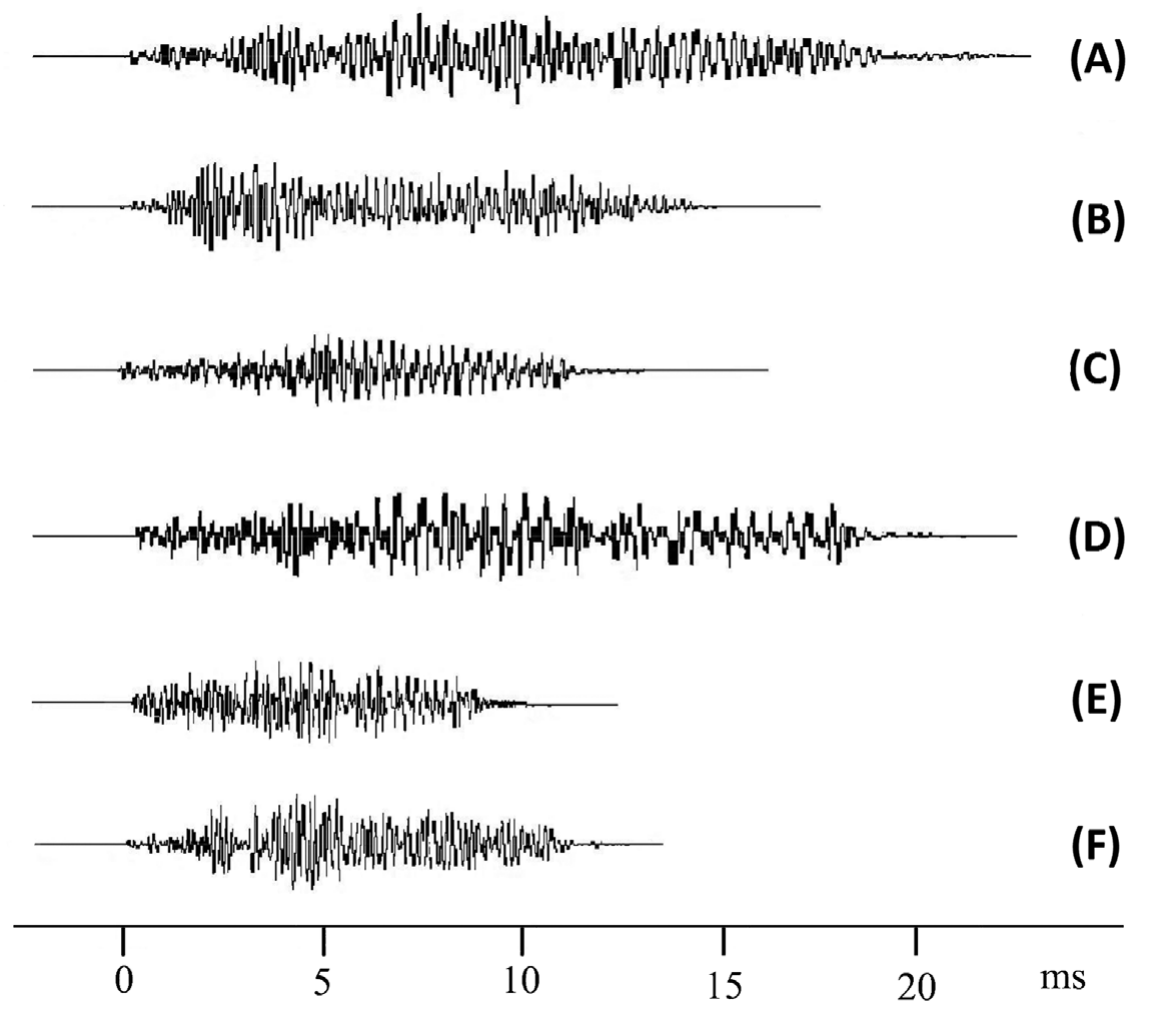
Presentation of one syllable of calling songs showed in Fig. 2 (**A-F**: CES, BYCG, JDM, WCG, SMJ and ELC).

**Figure 4.**
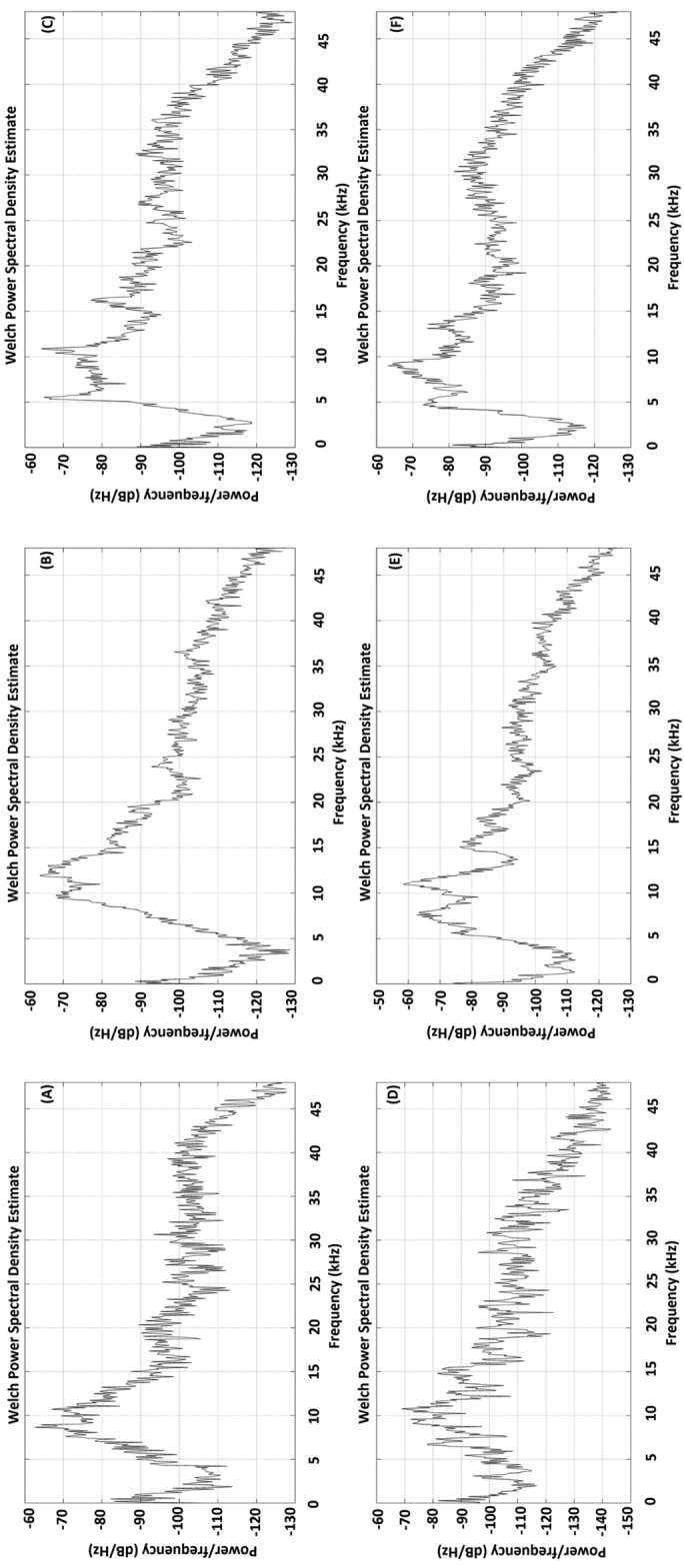
Power spectral density of the calling songs of *Gampsocleis sedakovii* from six geographic populations (**A–F**: CES, BYCG, JDM, WCG, SMJ and ELC).

Acoustic parameters measured are shown in Table 2. The calling song of the individuals of *Gampsocleis sedakovii* was continuous, consisting of series of single pulses (Fig. 2, 3). In addition, the power spectral density (PSD) was analyzed (Fig. 4). Analysis of variance showed that there were significant differences in all song features among the samples captured at different locations (Table 3), and the dissimilarity of samples between locations showed significantly different (Table 4).

**Table 2.** Time-domain and frequency-domain features of *Gampsocleis sedakovii* from six geographic populations.

Location	PD (ms)	PI (ms)	PRR	DF (kHz)	HF (kHz)	LF (kHz)	GAN
CES	20.4 ± 0.00	13.1 ± 0.00	0.028 ± 0.00	8.1 ± 0.10	23.7 ± 0.28	5.9 ± 0.09	KT283620 ~ KT283622
BYCG	13.3 ± 0.00	14.1 ± 0.00	0.036 ± 0.00	12.0 ± 0.19	21.0 ± 0.10	7.1 ± 0.09	KT283617 ~ KT283619
JDM	11.8± 0.00	10.8 ± 0.00	0.044 ± 0.00	10.8 ± 0.04	22.1 ± 0.07	4.9 ± 0.04	KT283614 ~ KT283616
WCG	20.1 ± 0.00	12.1 ± 0.00	0.031 ± 0.00	10.6 ± 0.03	19.3 ± 0.15	5.1 ± 0.06	KT283605 ~ KT283607
SMJ	9.4 ± 0.00	9.2 ± 0.00	0.054 ± 0.00	11.1 ± 0.06	19.6 ± 0.07	4.8 ± 0.05	KT283611 ~ KT283613
ELC	10.4 ± 0.00	10.0 ± 0.00	0.049 ± 0.00	8.3 ± 0.26	19.8±0. 23	5.1±0.06	KT283608 ~ KT283610

Abbreviation: PD : Pulse duration ; PI : Pulse interval ; PRR : Pulse repetition rate ; DF : Dominant frequency ; HF : Highest frequency ; LF : Lowest frequency ; GAN : GenBank accession number . Note: the acoustic data were obtained from five individuals from each site; the genetic data are from three individuals included in the acoustic study.

**Table 3.** Analysis of variance tables for the analysis of calling song and morphological traits for male *Gampsocleis sedakovii* among six geographic populations.

	Mean Square	d.f.	F	Sig.
PD	0.001	5	188.344	<0.001*
PI	0.000	5	61.899	<0.001*
DF	50.170	5	88.193	<0.001*
HF	113.971	5	1123.716	<0.001*
LF	22.599	5	127.105	<0.001*
WL	351.056	5	1129.041	<0.001*
WTSF	1041.250	5	6.818	<0.001*
NTSF	9.289	5	1.268	0.381
LP	98.797	5	2964.154	<0.001*
BL	1532.304	5	12162.586	<0.001*

* indicates a significant difference at the 0.05 level.

Abbreviations: PD , pulse duration ; PI , pulse interval ; DF , dominant frequency ; HF , highest frequency ; LF , lowest frequency ; WL , length of wing ; WTSF , width of tooth of a stridulatory file ; NTSF , number of teeth of a stridulatory file ; LP , length of pronotum ; BL , the body length .

**Table 4.** The proximity matrix of analysis of distance of these geographical populations.

	Euclidean Distance					
	CES	BYCG	JDM	WCG	SMJ	ELC
CES	.000	5.925	3.520	14.312	14.889	13.455
BYCG	5.925	.000	3.772	11.254	11.715	11.261
JDM	3.520	3.772	.000	13.255	13.751	12.876
WCG	14.312	11.254	13.255	.000	.950	2.436
SMJ	14.889	11.715	13.751	.950	.000	3.088
ELC	13.455	11.261	12.876	2.436	3.088	.000

Note: this dissimilarity matrix was obtained by all data including the acoustic, morphological, and genetic information.

### Morphological traits

**Figure 5.**
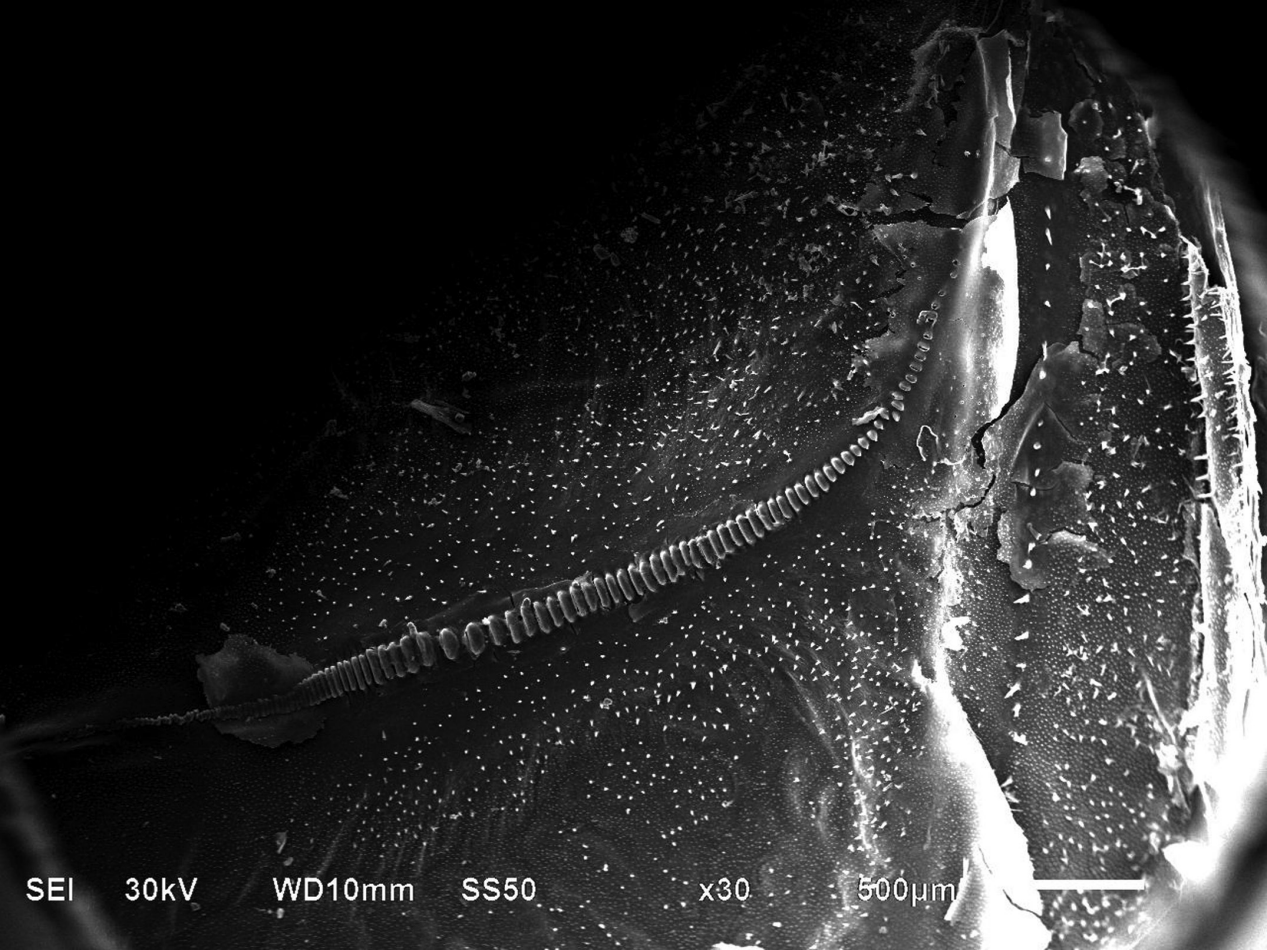
SEM of the stridulatory file of *Gampsocleis sedakovii*.

SEMs as used to determine if the stridulatory files of *Gampsocleis sedakovii* from specimens of different localities were similar to each other. They were claviform and the teeth in the middle section were wider than those located at both ends of the file (Fig. 5).

In this part of observation, all six morphological traits, except for the number of teeth of a stridulatory file, had significant differences among the other five morphological parameters across the individuals captured from six locations (Table 3 and 5).

**Table 5.** Morphological characteristics of specimens from the different sampling sites.

Location	CES	BYCG	JDM	WCG	SMJ	ELC
NTSF	116.7 ± 0.41	115.1 ± 0.49	115.5 ± 0.55	115.6 ± 0.47	115.3 ± 0.54	115.5 ± 0.49
WL (mm)	34.0 ± 0.12	34.0 ± 0.12	34.0 ± 0.12	27.1 ± 0.06	27.3 ± 0.06	28.3 ± 0.05
WTSF (µm)	93.0 ± 3.07	96.0 ± 1.16	93.5 ± 3.08	104.5 ± 0.62	105.2 ± 0.68	103.9 ± 0.44
LP (mm)	7.8 ± 0.02	6.8 ± 0.01	8.7 ± 0.02	8.3 ± 0.01	8.6 ± 0.02	8.5 ± 0.01
BL (mm)	29.1 ± 0.03	24.1 ± 0.04	31.5 ± 0.03	28.5 ± 0.01	33.0 ± 0.03	31.6 ± 0.03
WL/LP	4.1 ~ 4.4	4.8 ~ 5.2	3.7 ~ 4.1	3.1 ~ 3.2	3.1 ~ 3.3	3.3 ~ 3.6

Abbreviations: NTSF , The number of teeth of a stridulatory file ; WL , wing length ; WTSF , width of tooth of a stridulatory file ; LP , length of pronotum ; BL , body length . Note: The wing length was measured from end of the pronotum to the wing tip. The body length was measured from forehead to the end of abdomen.

### Sequence of mtDNA-COI

**Figure 6.**
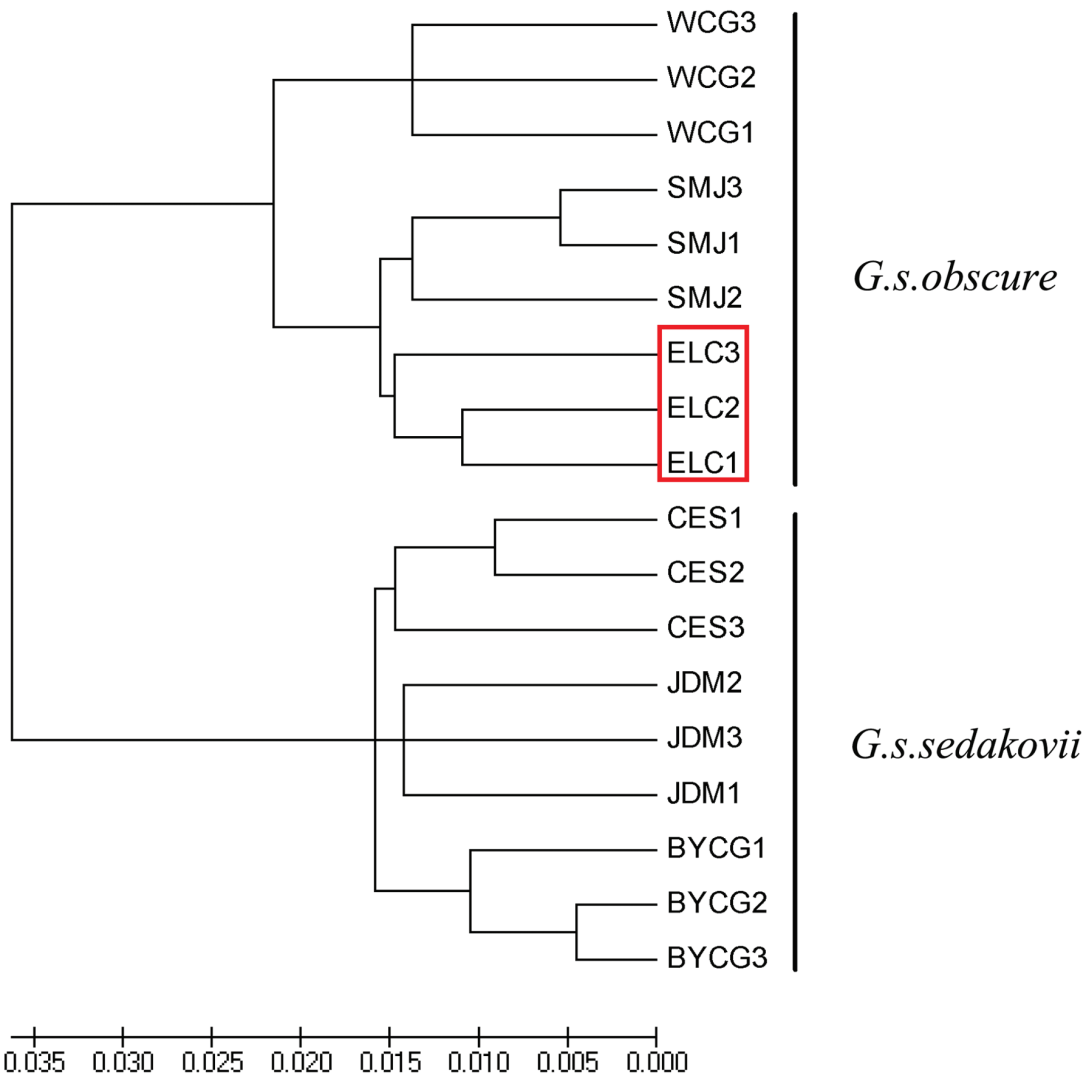
Neighbour-Joining (NJ) tree based on COI sequence from 18 individuals of *Gampsocleis sedakovii* collected from six sampling sites (CES, BYCG, JDM, WCG, SMJ, and ELC).

Based on the sequence of partial mtDNA (COI), individuals from six locations distinctly formed two separate clades in the NJ analysis. One clade consisted of the individuals from CES, BYCG, and JDM, while the individuals of the other three sites were grouped together (Fig. 6). Results suggested that there were some differentiations among these samples collected from different sites at the molecular level. The GenBank accession number is shown in Table 2.

### Cluster results

**Figure 7.**
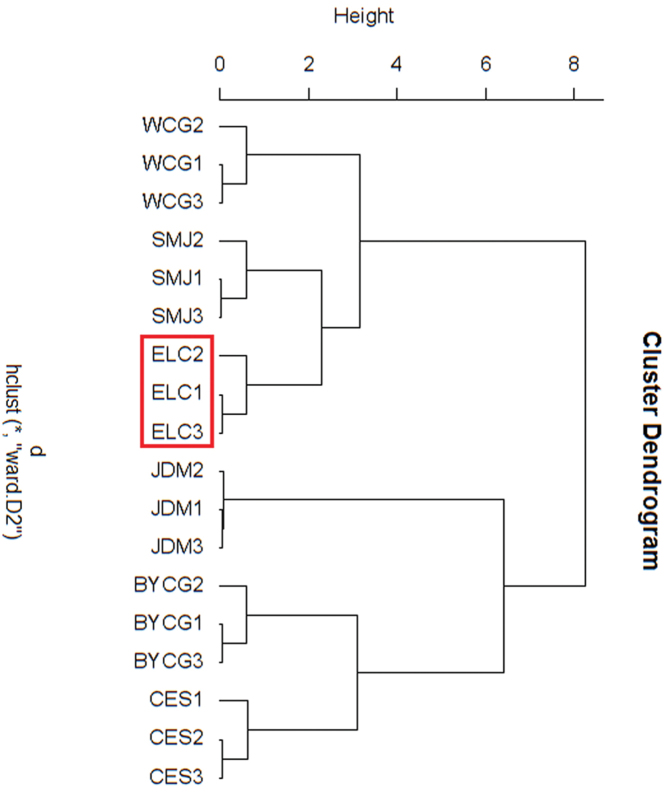
Dendrogram generated by cluster analysis based on acoustic characteristics.

**Figure 8.**
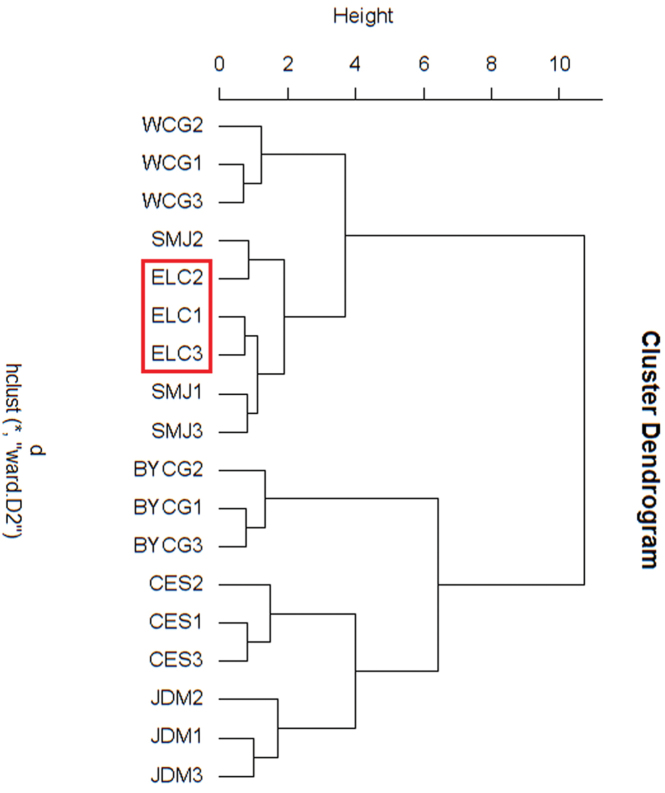
Dendrogram generated by cluster analysis based on morphological traits.

Based on five song traits and six morphological parameters, individuals from the six regions were clustered, based on acoustic traits and morphological parameters respectively, and it was found that the cluster results were consistent with each other. Both cluster results of acoustic signals and morphological features showed there were two main clades among these samples. Specifically, individuals from CES, BYCG, and JDM grouped together and composed one branch. The other branch consisted of the individuals from SMJ, WCG, and ELC (Figs 7 and 8). This result was in accordance with the molecular data. The clustering analyses using the three criteria of acoustics, morphology, and genetic analysis, all gave similar results. Interestingly, through these results, it was found that the *Gampsocleis sedakovii* from ELC had the median values of all three characteristics. Shown in Table 4, the dissimilarity matrix, the Euclidean distances of ELC were also in the middle.

## Discussion

In this study molecular, acoustic, and morphological differentiation has been analyzed in *Gampsocleis sedakovii* collected from six sampling sites. By genetic analysis, the individuals from different geographical populations grouped into two clades. This was consistent with the results from the analysis of calling songs and morphological characteristics. For *Gampsocleis sedakovii*, the morphological features were used to support traditional taxonomy. However, using only morphological traits led to different conclusions and using genetic data, Zhou et al. (2011) showed that the subspecies distinctions did not match precisely the differences in morphology. However, this research supports the view that there are two subspecies of *Gampsocleis sedakovii* based on morphological features, in accordance with the traditional classification.

In contrast with these results, the description of the songs of *Gampsocleis sedakovii sedakovii*, previously made by Wu and Shi (2009), showed that there were two kinds of chirps. We speculate this difference might be related to different sampling sites. The studied species used by Wu and Shi were collected from Hebei province, while we captured the *Gampsocleis sedakovii sedakovii* in Inner Mongolia area. Different calling songs for different locations might be the result of adaption to specific habitats.

Evolutionary studies of selected orthopteran taxa have improved our knowledge of the role that insect songs play in speciation (Shaw et al. 2007, Vedenina et al. 2007). The song differentiation of subspecies of *Gampsocleis sedakovii* remains unknown. Is there a difference between the two subspecies? How much difference was and the cause of this difference remained unknown until now. To increase our knowledge of the evolutionary mechanisms that generate song diversity and the process of subspeciation, it is crucial to study the songs of subspecies. We inferred that the katydids from ELC were the “intermediate type” of *Gampsocleis sedakovii*. No matter which criteria were applied for classification, these individuals remained intermediate. From the dissimilarity matrix, this phenomenon was also obvious. There were two groups (one for CES, JDM, and BYCG, called group one; the other for WCG, SMJ, and ELC, called group two) and as a whole the Euclidean distance between groups was bigger than within each group. What is noteworthy was that the distances between ELC and group one were smaller compared to the other two sites of group two. Therefore, we inferred it might be the transition to subspeciation. At the same time, we found that the calling songs changed gradually in the process of subspecies formation. In a previous study, the northeast region of China was thought to be the centre of differentiation of *Gampsocleis sedakovii* (see Bey-Bienko 1930). In the process of diffusion, evolution took the form of radiation, so we conclude that ELC was closer to the centre of differentiation.

In the study of *Apis cerana*, the discovery of the new species showed that the classification of subspecies need not be based on differences in geographical region (Zhuang 1989). However, although distributed in geographically close regions, individuals might belong to different subspecies.

In other animal groups, such as frogs (Amézquita et al. 2009, Funk et al. 2009, Velásquez et al. 2013), birds (Irwin et al. 2008), and some primates (Thinh et al. 2011, Meyer et al. 2012), positive correlations between bioacoustic traits and genetic differences have been reported. Jaiswara et al. (2012) showed that the phylogenetic analyses largely supported the acoustic clusters for the genus *Itaropsis*, and these two lineages were further supported with morphological variation. Our data supports the idea that the structure of acoustic signals is closely related to genetic differences among populations and provides some evidence that this relationship exists on the subspecies level.

In summary, this study shows that there are two lineages within the species *Gampsocleis sedakovii*. This conclusion supports the existing classification with two subspecies. Further examination, including samples from more geographical populations, will be needed for a more robust assessment of phylogenetic analysis.

## Conclusions

Two large groups within species *Gampsocleis sedakovii* were discovered by performing genetic, morphological, and acoustic analysis. Our data justifies the existing classification of *Gampsocleis sedakovii* into two subspecies, *Gampsocleis sedakovii sedakovii* and *Gampsocleis sedakovii obscura*. We found the calling songs differed with geographical distribution, suggesting that acoustic variation might play an important role in the formation of new subspecies.
